# Atrial Fibrillation in a Patient With an Accessory Pathway

**DOI:** 10.1177/2324709618802870

**Published:** 2018-09-28

**Authors:** Andrew Silverman, Sonia Taneja, Liliya Benchetrit, Peter Makusha, Robert L. McNamara, Alexander B. Pine

**Affiliations:** 1 Yale University, New Haven, CT, USA

**Keywords:** Wolff-Parkinson-White syndrome, WBW, atrial fibrillation, accessory pathway, preexcitation syndrome, electrophysiology, radiofrequency ablation

## Abstract

A 24-year-old man with history of unspecified arrhythmia presented with palpitations and chest pain. Initial electrocardiogram (ECG) revealed irregular tachycardia with varying QRS width: 150 to 200 beats per minute for narrow complexes and 300 beats per minute for wide complexes. Following cardioversion, ECG revealed sinus tachycardia with a preexcitation pattern of positive delta waves in the anterolateral leads and negative delta waves in inferior leads. The patient remained in sinus rhythm and underwent successful ablation of a right posteroseptal accessory pathway. Subsequent ECG showed upright T waves in the leads I, aVL, and V2-6, large inverted T waves in leads III and aVF, and no delta waves. This case serves as an important reminder that atrial fibrillation (AF) in the presence of an accessory pathway may present with confounding ECG features, potentially leading to incorrect diagnoses and treatments that may be life threatening. Despite 10% to 30% prevalence of AF in the presence of an accessory pathway and the relative awareness of Wolff-Parkinson-White syndrome among general internal medicine providers, the clinical recognition of Wolff-Parkinson-White syndrome may be hindered in the presence of preexcited AF.

## Case Report

**Figure 1.**
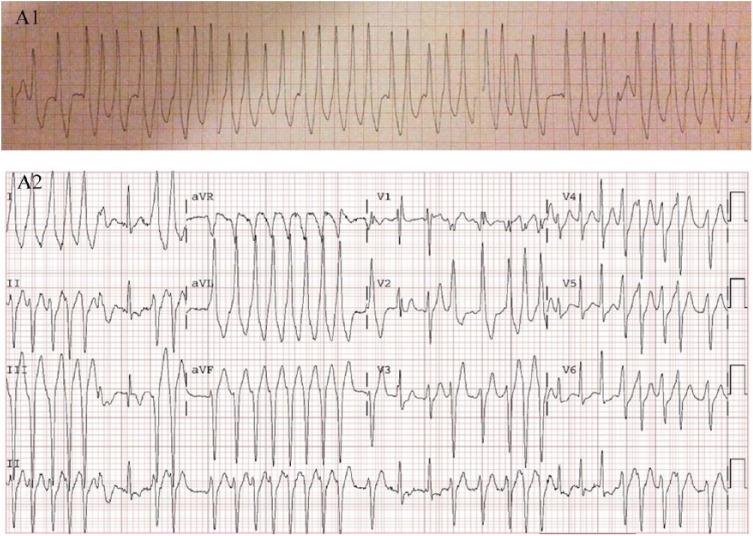
(A1) Initial strip en route to the hospital, revealing an irregular wide-complex tachycardia with varying QRS width and a ventricular rate up to 300 bpm; upstroke of some QRS complexes may appear slurred. (A2) First electrocardiogram in the emergency department showed an irregularly irregular tachycardia; QRS complexes reveal variable degrees of preexcitation.

A 24-year-old man with a history of unspecified intermittent arrhythmia presented with sudden-onset palpitations, sharp left-sided chest pain, left arm numbness, shortness of breath, lightheadedness, and a feeling of impending loss of consciousness. He described similar past episodes now occurring more frequently lasting several minutes and abating with deep breaths and “clenching up” the chest. The prehospital electrocardiogram (ECG) strip revealed an irregular wide-complex tachycardia (WCT) with varying QRS width and a ventricular rate up to 300 beats per minute (bpm). The upstroke of some QRS complexes appeared slurred (Figure 1-A1).

On arrival to the emergency department, his vitals included a heart rate greater than 200 bpm and a systolic blood pressure of 130 mm Hg. His oxygen saturation was 100% on room air. On examination, the patient was alert and oriented with an intact neurologic examination. His lungs were clear to auscultation bilaterally without wheezes, rhonchi, or rales. The cardiovascular examination was notable for tachycardia with an irregularly irregular rhythm. There were no extra heart sounds, including murmurs, rubs, and gallops. The abdomen was soft, nontender, and nondistended, and the extremities were warm and well perfused. He had strong palpable pulses in his hands and feet, and there was no lower extremity edema. The patient had no prior diagnosis of structural heart disease. He took no medications and had no known drug allergies. Both his family and social history were noncontributory to his current presentation.

**Figure 2.**
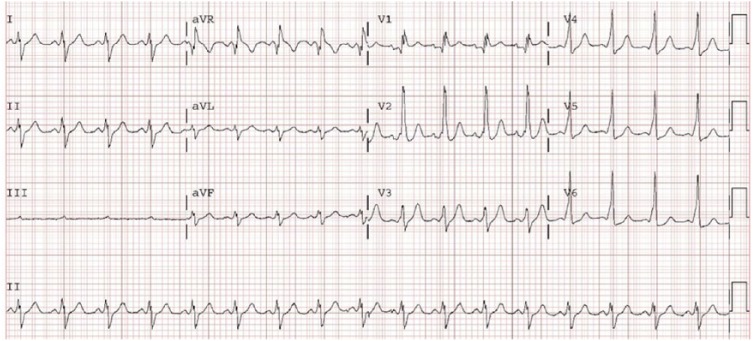
Electrocardiogram obtained following the cardioversion at 100J, showing sinus tachycardia as well as the preexcitation pattern with notable delta-waves.

The first 12-lead ECG acquired in the emergency department showed irregular tachycardia with polymorphic QRS complexes of varying width, along with several narrow normal-appearing complexes (Figure 1-A2). The heart rate demonstrated variable preexcitation with rates up to 300 bpm. Several wide QRS complexes in the lateral leads exhibited the slurred upstroke phase, which was not the case for the narrow complexes. The patient was fully awake with systolic blood pressures in 130s mm Hg. Because of the initial interpretation of the rhythm as ventricular tachycardia, the patient was given 2 rounds of amiodarone 150 mg intravenous without effect. He was then cardioverted with 100J, synchronized. The post-cardioversion ECG showed sinus tachycardia with the preexcitation pattern of positive delta waves in the anterolateral leads (I, aVL, and V2-6; Figure 2).

**Figure 3.**
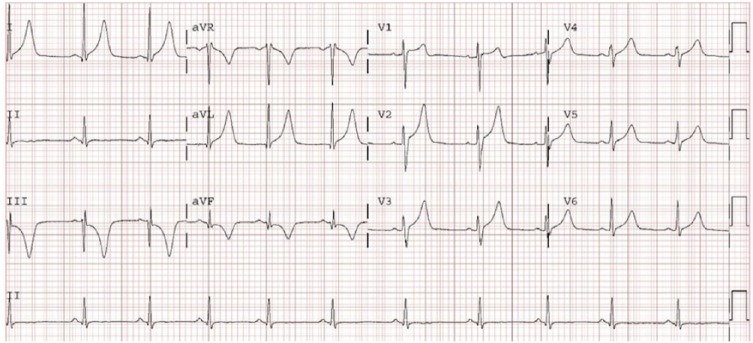
After successful ablation of the posteroseptal accessory pathway, the patient’s electrocardiogram no longer exhibited delta waves; peaked T waves can be observed in the inferior and anterior leads (classic post-ablation “pseudo inferior wall myocardial infarction” pattern), often considered as the evidence of a successful ablation.

The patient remained in sinus rhythm and was admitted to a medicine floor. The following day, he underwent successful radiofrequency ablation of a right posteroseptal accessory pathway. Subsequent ECG strips showed no delta waves but revealed peaked upright T waves in leads I, aVL, and V2-6, and large inverted T waves in leads III and aVF (Figure 3). The troponin level peaked at 0.53 ng/mL, and was undetectable within 12 hours. An echocardiogram was unrevealing. The patient was discharged after 3 days in stable condition remaining in normal sinus rhythm.

## Discussion

This case serves as an important reminder that atrial fibrillation (AF) in the presence of an accessory pathway may present with confounding electrocardiographic features, potentially leading to incorrect diagnoses and treatments that may be life threatening. Despite a 10% to 30% prevalence of preexcited AF in the presence of an accessory pathway^1^ and the relative awareness of Wolff-Parkinson-White (WPW) syndrome among general medicine providers, the clinical recognition of WPW may be hindered in the presence of preexcited AF. Indeed, in a survey study of emergency medicine physicians, this challenging tachyarrhythmia was identified as WPW syndrome by only 18% of responders, while less than 10% of participants identified AF.^2^

To review the classical manifestations of WPW syndrome, it is important to recall the presence of the bundle of Kent, otherwise referred to as the accessory pathway through which fast anterograde conduction can outpace slower atrioventricular (AV) node conduction. This pathway results in a relatively quick depolarization of the ventricles, resulting in distinct ECG changes like a short PR interval, wide QRS complex, and the virtually pathognomonic delta wave. Moreover, in “concealed” WPW syndrome, it is difficult to discern any electrocardiographic anomalies at baseline, as the accessory pathway may not conduct in an anterograde fashion.^3^ In the majority of WPW patients, paroxysmal AV reentrant tachycardia (AVRT) occurs via anterograde conduction through the AV node, followed by retrograde conduction through the bundle of Kent (orthodromic AVRT), producing a tachycardia with narrow QRS morphology. These patients typically do not demonstrate rapid preexcitation responses during AF, likely due to either anterograde conduction delay of the accessory pathway relative to the AV node or block. Wide QRS tachycardia, in contrast, can occur in patients with antidromic AVRT, whereby anterograde conduction through the accessory pathway is followed by retrograde conduction through the AV node. This circuit may also transpire in patients with preexisting bundle branch blocks.^4,5^

During preexcited AF, the atria can discharge at a rate higher than 300 impulses per minute, obscuring delta waves—the key electrocardiographic feature of WPW syndrome. The AV node normally blocks most of these impulses due to decremental conduction, an intrinsic repolarization property that allows the node to conduct more slowly when it receives faster signals. However, an accessory pathway without such a built-in delay makes 1:1 conduction possible, with ventricular rates reaching 300 bpm. Preexcited AF is thus characterized as a malignant arrhythmia, as sudden cardiac death may result from this rhythm degenerating into ventricular fibrillation.^5,6^

In our case, the patient’s initial ECG reflected irregular chaotic WCT with varying morphology and width, partly because of abnormal depolarization along the accessory pathway. It was also apparent that some impulses were conducted through the AV node, as evidenced by narrow QRS complexes without the delta wave. Since the impulses travel through both the AV node and accessory pathway, treatment with AV nodal blockers (eg, adenosine, calcium channel blockers, β-blockers, and possibly amiodarone) is contraindicated because atrial impulses would then preferentially conduct through the accessory pathway in an antidromic direction.^7-9^ This can cause the rhythm to degenerate further into ventricular fibrillation, a life-threatening rhythm. On the other hand, in patients with narrow QRS AVRT, AV nodal blockers like adenosine or diltiazem are first-line agents; blocking the orthodromic reentrant circuit in these cases interrupts the tachycardia and can restore sinus rhythm. Vagal maneuvers function in a similar fashion.

Thus, the key to recognition of WPW syndrome with preexcited AF is the irregular WCT with QRS of varying morphology and amplitude with sustained rates exceeding 200 bpm. If the patient’s blood pressure is stable, either procainamide or ibutilide may be effective in slowing conduction velocity of the accessory pathway. This rhythm can be difficult to differentiate from polymorphic ventricular tachycardia, but the immediate treatment for both, in the context of hemodynamic instability, is electrical cardioversion. For prevention of recurrent arrhythmias, the definitive treatment for preexcited AF in WPW syndrome is radiofrequency ablation.

The final teaching point is to appreciate that following ablation large peaked T waves may appear in leads where the delta wave was most noticeable, with the concordant polarity. Namely, in leads where the delta wave was positive (leads I, aVL, and V2-6 in our patient), T waves are positive as well. Such an ECG abnormality is a classic post-ablation memory T wave pattern, often considered evidence of a successful ablation.^10^
